# Restricted streptomycin use in apple orchards did not adversely alter the soil bacteria communities

**DOI:** 10.3389/fmicb.2013.00383

**Published:** 2014-01-31

**Authors:** Fiona Walsh, Daniel P. Smith, Sarah M. Owens, Brion Duffy, Jürg E. Frey

**Affiliations:** ^1^Federal Department of Economic Affairs, Education and Research EAER, Research Station Agroscope Changins-Wädenswil ACWWädenswil, Switzerland; ^2^Institute of Genomic and Systems Biology, Argonne National LaboratoryArgonne, IL, USA; ^3^Computation Institute, University of ChicagoChicago, IL, USA; ^4^Environmental Genomics and Systems Biology Research Group, Institute for Natural Resource Sciences, LSFM, Zurich University of Applied SciencesZurich, Switzerland

**Keywords:** *Pseudomonas*, QIIME, CatchAll, 16S rRNA, *Burkholderia*

## Abstract

Streptomycin has been authorized for restricted use in the prevention of the fire blight disease of pome fruit orchards in the EU and Switzerland. This study addresses the important topic of the influence of the use of streptomycin in agriculture on the total bacteria community within the soil ecosystem. Soil samples were taken from soils under apple trees, prior to streptomycin application and 2 weeks post streptomycin application or water application (untreated control). High throughput 16S rRNA gene amplicon sequencing was used to generate datasets from the soils under apple trees in apple orchards from three different locations in Switzerland. We hypothesized that the use of streptomycin would reduce the bacterial diversity within the soil samples and enhance a reduction in the variety of taxa present. Bacterial species such as *Pseudomonas*, *Burkholderia*, and *Stenotrophomonas* are intrinsically resistant to many antibiotics and as such it is of interest to investigate if the use of streptomycin provided a selective advantage for these bacteria in the soil ecosystem. The application of streptomycin did not influence the abundance and diversities of major bacteria taxa of the soils or the *Pseudomonas*, *Burkholderia*, and *Stenotrophomonas* species. We also discovered that apple orchards under the same management practices, did not harbor the same bacterial communities. The restricted application of streptomycin in the protection of apple orchards from the fire blight pathogen *Erwinia amylovora* under the guidelines in Switzerland did not alter either the bacterial diversity or abundance within these soil ecosystems.

## Introduction

Antibiotics are used in plant agriculture for the control of plant pathogenic bacteria and, although resistance selection has only been found in the target phytopathogens (Stockwell and Duffy, 2012), they have been suggested a potential factor in the spread of antibiotic resistance within the food chain to human pathogens (Vidaver, 2002). Streptomycin was first used in plant agriculture in the 1950s and has been used since then in the prophylactic treatment of fire blight disease in apple and pear orchards. It is also registered for the control of fire blight in Israel, New Zealand, Canada, and Mexico (Stockwell and Duffy, 2012). Fire blight is a destructive bacterial disease of apple and pear trees caused by *Erwinia amylovora* and streptomycin remains the most reliable and commercially effective control product available against fire blight (Norelli et al., 2003).

Due to the perceived health and environmental risks associated with the use of antibiotics in agriculture, the use of streptomycin in plant agriculture was restricted within the EU in 2004 (Phillips, 2007). The seminal findings of Aarestrup (1995) in the identification of a link between the use of avoparcin as an animal growth promoter and increased vancomycin resistance highlighted the potential link between the use of antibiotics in agriculture and the selection for resistance (Aarestrup, 1995). The application of manure from animals treated with antibiotics as fertilizer has been linked to an increase in the abundance and diversity of antibiotic resistance genes in the environment (Binh et al., 2008). However, there is little knowledge on the ecological impact of antibiotic use in plant agriculture on the total soil bacterial populations.

The application of antibiotics in agriculture has been postulated to alter the overall microbial populations in natural ecosystems, which is of particular concern regarding microorganisms that co-colonize environmental and human ecosystems (Garmendia et al., 2012). Bacteria are essential components of a productive and healthy soil ecosystem and have many important known functions including decomposition, nutrient cycling, and protection against plant disease, thus it is important to identify the impacts of antibiotic application on their community composition (Brussaard et al., 1997). We hypothesized that the use of streptomycin would reduce the bacterial diversity within the soil samples and result in a reduction in the variety of taxa present. The multi-drug resistant hospital acquired pathogens such as *Burkholderia* species, *Stenotrophomonas* species, and *Pseudomonas aeruginosa* reside naturally in the soil environment (Walsh and Duffy, 2013). As these bacteria are intrinsically resistant to many antibiotics it was of interest to investigate if the use of streptomycin provided a selective advantage for these bacteria in the soil. This study utilized 16S rRNA amplicon meta sequencing to compare the effects of the use of streptomycin in plant agriculture on bacterial populations within soil ecosystems of comparable, replicated streptomycin treated, and control apple orchards.

## Materials and methods

### Sampling and site descriptions

The geographical and descriptive characteristics of the analyzed agricultural soil samples are described in Table 1. Nine soil samples were taken in total, comprising one sample from each orchard in 2008, prior to treatment, one sample from each orchard of a row of apple trees 2 weeks after being sprayed with streptomycin and one sample from each orchard of a row of apple trees 2 weeks after being sprayed with water. Soil samples consisted of eight soil cores (10 cm depth) per replicate taken using a stainless steel corer with an internal diameter of 2.5 cm. Soil cores were pooled for each replicate in the field. Pooling of soil cores is standardly applied in order to obtain more representative samples for a certain field plot or a specific experimental treatment (Milling et al., 2004). Eight separate soil core replicates were taken per sample, each set of eight soil cores were combined to represent the entire row of trees within an orchard under either streptomycin or water treatment at each of the time points. The sample in 2008 were collected on May 21st. The orchards were sprayed with streptomycin or water in 2011 on the 15th, 20th, and 27th of April and the samples were taken on the 11th of May. The soil pH was determined by suspending 1 g of soil in 2.5 mL 0.01 M CaCl_2_ and measuring the pH using a glass electrode (Will et al., 2010).

**Table 1:** **Geographical and descriptive characteristics of the analyzed orchard soil samples**.

**Sample ID**	**Date of isolation**	**Apple orchard**	**Elevation (m)**	**Latitude**	**Longitude**
FWS5	21.05.2008	Güttingen	503	47.6	9.2833
FW360 (S)	11.05.2011	Güttingen	503	47.6	9.2833
FW339 (W)	11.05.2011	Güttingen	503	47.6	9.2833
FWS4	21.05.2008	Lindau	485	47.4433	8.6664
FW321 (S)	11.05.2011	Lindau	485	47.4433	8.6664
FW330 (W)	11.05.2011	Lindau	485	47.4433	8.6664
FWS1	21.05.2008	Wädenswil	407	47.24368	8.78837
FW309 (W)	11.05.2011	Wädenswil	407	47.24368	8.78837
FW312 (S)	11.05.2011	Wädenswil	407	47.24368	8.78837

S, streptomycin treated orchard; W, water treated orchard.

### Orchard descriptions

**Figure 1:**
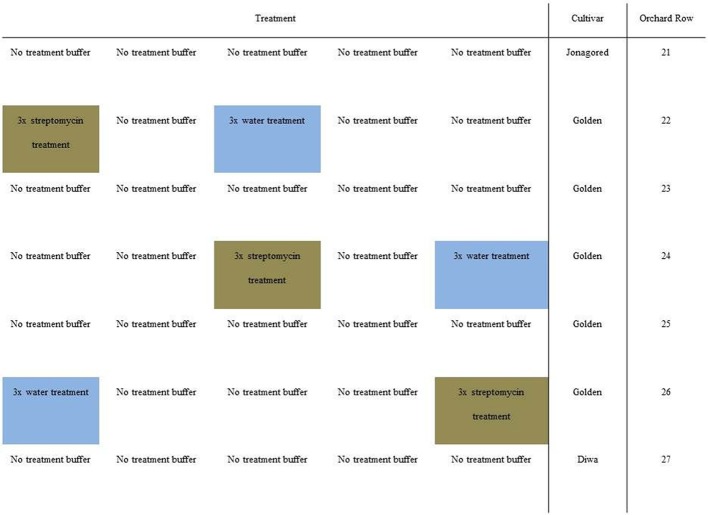
**Schematic diagram of the three orchard sites and streptomycin or water spraying strategies**.

Three apple orchards in Wädenswil, Lindau, and Güttingen in Switzerland were used for these experiments. These apple orchards were not used for commercial apple production, but were specifically used for research purposes. Each orchard consisted of treatment plots i.e., streptomycin or water, separated by non-treated buffer rows of apple trees (Figure 1). All trees were “Golden Delicious” trees. Applications of streptomycin at standard agricultural rates were scheduled based on apple flowering phenology with three applications. Normally, application is timed-to and limited-to potential infection conditions using forecasting models. Streptomycin formulation (600 g ha ^−1^) (Strepto, W 6528, Schneiter AGRO AG) was applied using low-drift spraying equipment.

### Calculation of the worst case scenario concentration of streptomycin in the soil

The amount of streptomycin sprayed was 600 g/ha, which is 60 mg/m^2^ and 60 μg/cm^2^. The soil sample taken was a cylinder of approximately 49 cm^3^, the top side (directly exposed to streptomycin) having an area of 4.9 cm^2^, meaning an average concentration of (60 × 4.9)/49 = 6 μg/cm^3^ soil, assuming an even distribution of streptomycin along the depth of 10 cm. In reality, there probably will be a concentration gradient from top to bottom. The spraying was performed twice, thus the highest possible concentration of streptomycin applied to the soil samples was 12 mg/L.

### DNA extraction, amplification of 16S rRNA genes, and library generation

The 16S rRNA gene amplification and next-generation sequencing was performed as part of the Earth Microbiome Project (EMP) (Bunge, 2009). DNA was extracted from each sample using the MoBio™ Power Soil^®^ DNA isolation kit (Süd-Laborbedarf GmbH, Gauting, Germany) and modifications according to the EMP protocols (Gilbert and Meyer, 2012). The primers for the paired-end 16S rRNA community sequencing were 515F (5′ GTGCCAGCMGCCGCGGTAA 3′) and 806R (5′ GGACTACVSGGGTATCTAAT 3′) (Caporaso et al., 2012). This primer set is reported to amplify nearly all bacterial taxa with few biases (Caporaso et al., 2012). The sequencing conditions and parameters are described on the EMP website (http://www.earthmicrobiome.org/emp-standard-protocols/).

### Statistical analysis of 16S rRNA amplicon sequencing data

Following sequencing on the Illumina MiSeq platform, the forward and reverse reads were joined using fastq-join from the ea-utils software package (Bergmann et al., 2011). The collection of 253-bp sized sequences were then quality filtered to exclude sequences with ambiguous (N) bases and sequences containing base calls with less than 99% confidence (Phred score of 20). Sequences passing these filters were then demultiplexed based on exact matches to 12-bp DNA barcode sequences incorporated during the PCR amplification step. The QIIME bioinformatics software suite was used to subsample the resultant 105,790 sequences to an even sampling depth of 4000 reads per sample, cluster those sequences into 97% identity operational taxonomic units (OTU) with UCLUST (Aronesty, 2011), taxonomically classify each OTU from RDP classifier (Edgar, 2010) placement into the GreenGenes 12_10 database (DeSantis et al., 2006), and generate the figures included with this report. All QIIME scripts were from the 1.5.0 release and run using default parameters unless otherwise stated.

In biology it is common to collect a sample of organisms and sort them into taxa. In the CatchAll software program the term “species” is used for these taxa, recognizing that this may not be exact in some cases. The estimated total diversity of “species” was determined using CatchAll (Bunge, 2009). CatchAll computes several different estimates and returns a ranked comparison of the “best” analyses for a given dataset. Data distribution was analyzed using the Student's *T*-Test corrected for multiple comparisons using the Benjamini–Hochberg procedure described below to investigate relationships between sample site locations, land use, pH, and anthropogenic effects such as antibiotic treatment. *P*-Values were calculated using a two-tailed Student's *T*-Test based on the abundances of each phylogenetic group among samples. *Q*-values were calculated via the Benjamini–Hochberg method to correct for the false discovery rate arising from multiple comparisons by iterating over thousands of OTUs and taxa (Benjamini and Hochberg, 1995). In this procedure, each *p*-value was multiplied by the number of phylogenetic groups examined, *T*, and then divided by that *p*-value's rank, *k*, among the set of *p*-values (e.g., the most significant *p*-value was divided by 1, the second most significant *p*-value was divided by 2, etc).

q-value=p-value×T/k

Only taxa abundance differences with a *q*-values of less than or equal to 0.05 were considered significant.

## Results and discussion

We compared and assessed the bacterial biodiversity in nine soils sampled from apple orchard soil ecosystems (Table 1). The soils were sampled prior to streptomycin use in 2008 (*n* = 3), in 2011 after streptomycin use (*n* = 3) and in 2011 after being sprayed with water (*n* = 3).

### Bacterial abundance and diversity richness

The 16S amplicon sequencing analysis using primers to amplify the 515–806 nucleotide, V4 region, of the 16S rRNA gene is well-suited for accurate placement of bacterial species (Liu et al., 2007). We obtained a total of 105,790 quality bacterial sequences, with a median amplicon length of 253 bp and a range of between 4134 and 9611 amplicons per sample. One of the statistical challenges for microbiome studies are to estimate population richness and diversity, model community structure, quantify uncertainty and compare estimates rigorously (Caporaso et al., 2010). The richness estimates were calculated using CatchAll to estimate the minimum number of different “species” in each population (Table 2). CatchAll statistical analyses defines the observed bacterial population as that which would be observed if the sampling and analysis protocols were to be performed to infinite effort (Bunge et al., 2014).

**Table 2:** **CatchAll statistical analysis of bacterial 16S sequence diversity data**.

**Sample ID**	**Orchard**	**Date of isolation**	**Tau**	**Observed Sp**	**Estimated total Sp**	***SE***	**Lower CB**	**Upper CB**	**GOF0**	**GOF5**
FWS4	Lindau	21.05.2008	11	1774	18,027	3355	12,672	26,041	0.0014	0.0538
FWS330 (W)	Lindau	11.05.2011	10	2472	25,832	4802	18,161	37,292	0.0007	0.0195
FWS321 (S)	Lindau	11.05.2011	14	2034	17,124	2392	13,119	22,589	0.0067	0.0293
FWS5	Güttingen	21.05.2008	10	2470	32,694	8160	20,453	53,322	0.0019	0.0432
FWS339 (W)	Güttingen	11.05.2011	51	2263	26,338	7310	15,716	45,350	0	0.0064
FWS360 (S)	Güttingen	11.05.2011	12	2942	34,112	4590	26,337	44,480	0.0003	0.0232
FWS1	Wädenswil	21.05.2008	10	2227	28,844	4929	20,802	40,376	0.0003	0.0127
FWS309 (W)	Wädenswil	11.05.2011	11	2762	48,611	15,414	26,918	89,855	0.0043	0.0444
FWS312 (S)	Wädenswil	11.05.2011	9	2682	59,646	32,370	22,926	163,437	0.0003	0.0136

The results described illustrate the variations in observed and estimated “species” within each soil. This provides an analysis of the variations in diversity and estimated diversity.

S, streptomycin treated; W, water treated.

Tau: A tau test is a non-parametric hypothesis test for statistical dependence based on the tau coefficient to measure rank correlation.

Estimated total species: estimated total diversity, which was calculated using the CatchAll model to identify the estimated number of “species” that could be present in the soil sample.

Sp, “species”; SE, standard error; Lower and upper CB, lower and upper 95 confidence bounds, respectively.

GOF0 should be regarded as a diagnostic\ divergence statistic, signaling divergence from the null hypothesis (which states that the model is correct), while GOF5 represents the p-value from a legitimate statistical hypothesis test of model.

The model used was the Two Mixed Experiment model for all except sample FWS339, where the Three Mixed Experiment model was used. The Two Mixed Model and Three Mixed Models are statistical sampling models used to analyze the data.

The CatchAll analyses indicated that the estimated target population diversity of each sample was higher than the observed population diversity. The number of “species” we observed from our finite collection of reads is likely to only represent a subset of the “species” present in the environment. The robustness of the data was confirmed by CatchAll as the GOF5 value for all of the samples was ≥0.01. The GOF5 value represents the *p*-value from a statistical hypothesis test of model fit. Smaller *p*-values represent evidence against the fit of the model, and larger *p*-values represent evidence in favor (Bunge, 2009). The difference between the observed and estimated richness highlights the vast array of bacterial species in soil. Similar orders of magnitude of difference between the observed and the estimated richness were observed in the analysis of phage metagenomic diversity data (Bunge et al., 2012). The estimated populations ranged from 17124 to 59646 species. The variations between the estimated populations were not associated with observed numbers of species or the treatment (with or without streptomycin). In soil FWS321 a large number of singletons were observed (*n* = 277) and a small number of very abundant species, indicating a large number of rare species in this soil.

### Alpha- and beta-diversity

**Figure 2:**
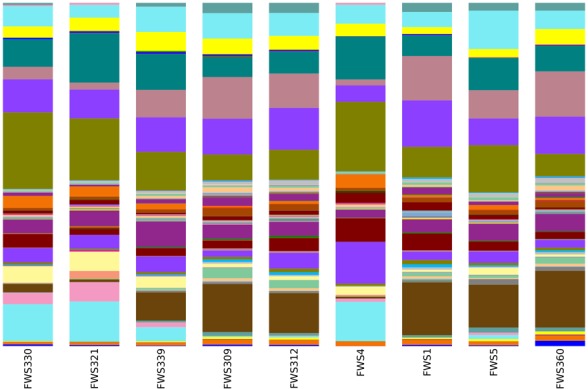
**Bacterial and archaeal community relative abundances of the major bacterial classes in soils derived from the sampling sites**. 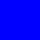: Archaea, Crenarchaeota, Thaumarchaeota; 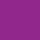: Bacteria, Acidobacteria, Other; 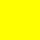: Bacteria, Acidobacteria; 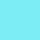: Bacteria, Acidobacteria, Acidobacteria; 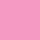: Bacteria, Acidobacteria, Acidobacteria-2; 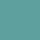: Bacteria, Acidobacteria, Acidobacteria-5; 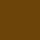: Bacteria, Acidobacteria, Acidobacteria-6; 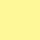: Bacteria, Acidobacteria, Solibacteres; 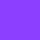: Bacteria, Actinobacteria, Actinobacteria; 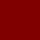: Bacteria, Actinobacteria, Thermoleophilia; 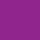: Bacteria, Bacteroidetes, Sphingobacteriia; 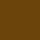: Bacteria, Chloroflexi, Other; 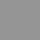: Bacteria, Chloroflexi, Anaerolineae; 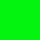: Bacteria, Chloroflexi, Bljii12; 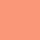: Bacteria, Chloroflexi, Chloroflexi; 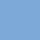: Bacteria, Chloroflexi, Ellin6529; 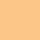: Bacteria, Chloroflexi, Ktedonobacteria; 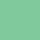: Bacteria, Chloroflexi, S085; 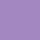: Bacteria, Chloroflexi, TK17; 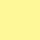: Bacteria, Chloroflexi, Thermomicrobia; 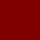: Bacteria, Firmicutes, Bacilli; 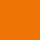: Bacteria, Gemmatimonadetes, Gemmatimonadetes; 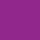: Bacteria, Nitrospirae, Nitrospira; 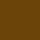: Bacteria, Planctomycetes, Other; 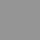: Bacteria, Planctomycetes; 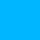: Bacteria, Proteobacteria, Other; 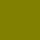: Bacteria, Proteobacteria, Alphaproteobacteria; 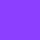: Bacteria, Proteobacteria, Betaproteobacteria; 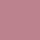: Bacteria, Proteobacteria, Deltaproteobacteria; 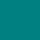: Bacteria, Proteobacteria, Gammaproteobacteria; 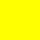: Bacteria, Verrucomicrobia, Pedosphaerae; 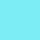: Bacteria, Verrucomicrobia, Spartobacteria.

The same bacterial phyla were identified in all soils, regardless of the influence of streptomycin (Figure 2 and Table 3). The most recent version of Greengenes has more than doubled in size from 408,000 sequences in the previous version to over 1 million (http://qiime.wordpress.com/). The bacterial diversity was examined at descending bacterial classification and using statistical analyses in order to identify differences in the abundances of different phyla. The most abundant phyla were consistent across all samples and consisted of Proteobacteria, Acidobacteria, Verrucomicrobia, Actinobacteria, Bacteroidetes, Gemmatimonadetes, and Planctomycetes (Figure 2 and Table 2). Proteobacteria were the most abundant phyla with abundance values ranging from 36.3% of the FWS309 soil population (Wädenswil orchard treated with water), to 43.7% in the soil FWS330 population (Lindau orchard treated with water). Acidobacteria were the second most abundant phyla in all soils. The Acidobacteria compositions of the soil populations comprised between 19.8 and 26.8% in the soils FWS312 (Wädenswil orchard treated with streptomycin) and FWS321 (Lindau orchard treated with streptomycin), respectively. The third most abundant phyla was Verrucomicrobia for all soils except FWS1, where Actinobacteria were then third most abundant. The percentage of population comprising Verrucomicrobia for all soils ranged from 6.8% in FWS1 to 13.7% in FWS5 and FWS339, water treated orchards from Wädenswil and Güttingen, respectively and averaged 10.7% for all soils. The results of this study are in contrast to studies of grassland and cultivated soils in Germany, where the Verrucomicrobia were between 0 and 3.4% (Milling et al., 2004; Shange et al., 2012). However, the Verrucomicrobia phyla have been underrepresented in many studies, due to biases in the PCR primers targeting 16S rRNA genes. Actinobacteria represented between 5.4% in soil FWS360, (streptomycin treated Güttingen) and 10% in soil FWS312, (streptomycin treated Lindau).

**Table 3:** **Taxonomy and percentage of the bacterial phyla population abundances of the bacterial 16S rRNA gene sequences from orchard soils treated with streptomycin in comparison to those treated with water in descending order of percentage of the population**.

**Taxonomy**	**Lindau apple orchard**			**Güttingen apple orchard**			**Wädenswil apple orchard**		
	**FWS4 water %**	**FWS330 water %**	**FWS321 strep %**	**FWS5 water %**	**FWS339 water %**	**FWS360 strep %**	**FWS1 water %**	**FWS309 water %**	**FWS312 strep %**
Bacteria; Proteobacteria	39.9	43.7	42.9	39.3	39.9	38.3	41.8	36.3	37.4
Bacteria; Acidobacteria	21.9	22.5	26.8	22.2	19.9	24.8	22.0	23.4	19.8
Bacteria; Verrucomicrobia	10.6	9.6	8.1	13.7	13.7	10.2	6.8	12.6	11.2
Bacteria; Actinobacteria	8.8	8.9	6.1	6.9	7.6	5.4	9.0	5.6	10.0
Bacteria; Bacteroidetes	4.8	4.2	4.9	5.2	7.8	5.2	4.1	4.6	4.7
Bacteria; Gemmatimonadetes	3.3	4.2	3.8	2.8	2.7	3.0	2.3	3.0	2.8
Bacteria; Planctomycetes	2.6	0.9	1.1	2.5	2.4	4.5	2.6	4.6	3.8
Bacteria; WS3	1.7	0.6	0.0	2.3	1.1	2.3	2.7	3.0	2.9
Bacteria; Firmicutes	1.5	1.0	1.4	1.4	0.5	0.6	2.4	1.2	1.3
Bacteria; Nitrospirae	1.3	1.0	0.5	1.0	1.2	1.1	1.9	2.6	2.1
Bacteria; Chloroflexi	1.0	1.1	0.7	0.7	1.3	0.9	1.7	0.6	1.0
Bacteria; Other	1.2	0.7	0.9	1.1	0.9	1.6	1.4	1.4	1.4
Archaea; Crenarchaeota	0.4	0.5	0.3	0.2	0.2	1.6	0.4	0.4	0.3
Bacteria; WPS-2	0.2	0.4	0.5	0.0	0.0	0.0	0.0	0.0	0.0
Bacteria; Armatimonadetes	0.1	0.1	0.5	0.1	0.0	0.0	0.0	0.0	0.0
Bacteria; Chlamydiae	0.1	0.1	0.3	0.2	0.1	0.0	0.3	0.0	0.0
Bacteria; Elusimicrobia	0.2	0.1	0.3	0.1	0.2	0.2	0.1	0.2	0.4
Bacteria; Chlorobi	0.2	0.1	0.1	0.0	0.2	0.2	0.3	0.2	0.3
Bacteria; Cyanobacteria	0.2	0.2	0.1	0.3	0.2	0.0	0.2	0.0	0.1
Bacteria; FCPU426	0.1	0.0	0.3	0.0	0.0	0.0	0.0	0.1	0.0
Bacteria; TM6	0.1	0.0	0.1	0.1	0.0	0.0	0.0	0.1	0.1
Unclassified; Other	0.0	0.0	0.0	0.0	0.0	0.0	0.0	0.0	0.0
Bacteria; AD3	0.0	0.0	0.1	0.0	0.0	0.0	0.0	0.0	0.0

Strep, streptomycin treated orchard; Water, water treated orchard.

The abundances of the most dominant phyla Acidobacteria, Proteobacteria, Verrucomicrobia, Actinobacteria, and Bacteroidetes, representing 86% of all bacteria on average, differ from a previous study, in which Acidobacteria, Actinobacteria, Proteobacteria, Bacteroidetes, and Firmicutes accounted for more than 90% of the sequences in the soils examined. In our study there us a lack of Firmicutes and presence of Verrucomicrobia. While Firmicutes were detected in the soils in our study, they were not represented at the same level of abundance as in previous studies.

### Statistically significant variations in bacterial abundance

The results from Lindau, Güttingen, and Wädenswil soils were compared to one another e.g., Lindau vs. Güttingen, Lindau vs. non-Lindau, etc.—to identify statistically significant variations in the taxa abundances (Table S1 in Supplementary Material). The *p*-value was calculated using a two-tailed Student's *t*-test based on the sets of abundances. The “PctChange” column in Table S1 in Supplementary Material displays the change in abundance as a percentage of the total bacterial population. There were no significant increases in the abundance of the intrinsically resistant bacteria belonging to the *Pseudomonas*, *Burkholderia,* and *Stenotrophomonas* species associated with the use of streptomycin. There were statistically significant higher abundances of the Acidobacteria Group 6 (11% change), Acidobacteriales (10% change), Acidobacteria (10% change), Alphaproteobacteria (9% change), Deltaproteobacteria (8% change), Koribacteraceae (5% change), Syntrophobacterales (5% change), Acidobacteriaceae (4% change), and Alphaproteobacteria; Ellin 329 (4% change) in the Lindau orchard soils in comparison to the non-Lindau orchard soils. There were also statistically significant differences in the bacteria taxa between the Lindau orchard soils and the Wädenswil orchard soils: Acidobacteriales (11%) and Acidobacteria (11%) were more abundant in the Lindau orchard. The differences in taxa abundance are illustrated in Figure 2, where the differences in the Acidobacter Group 1 and Acidobacter Group 6 are apparent as well as differences in abundance of Alphaproteobacteria. Differences in other bacteria abundances are visible but are not statistically significant e.g., Betaproteobacteria and Gammaproteobacteria.

The worst case scenario concentration of streptomycin that could have been applied to the soil was 12 mg/L, (two sprays at a final concentration of 6 mg/L each). The breakpoint concentrations of clinically relevant pathogens according to the CLSI guidelines are 16 mg/L or greater for *Yersinia pestis*, 8 mg/L or greater for *Brucella* species and *Francisella tularensis* (Clinical and Laboratory Standards Institute, 2006). The acceptable ranges of minimum inhibitory concentration (MIC) for the control strains *Staphylococcus aureus* ATCC 29213 and *Escherichia coli* ATCC 25922 are between 8 and 32 mg/L (Clinical and Laboratory Standards Institute, 2006). Thus, the final concentration of 12 mg/L could have inhibited the growth of selected clinically relevant bacteria. The acceptable range of MIC for the control strain *Pseudomonas aeruginosa* ATCC 27853 is between 32 and 128 mg/L (Clinical and Laboratory Standards Institute, 2006). Therefore, the worst case concentration of 12 mg/L would not have inhibited clinical *P. aeruginosa*. Caution must be taken in extrapolating the impact of streptomycin on soil bacterial population based on the clinically relevant breakpoints of selected bacteria. We do not know the natural concentration of streptomycin produced by the soil bacteria Actinomycetes in these soils. We also do not know what amount of the streptomycin is bound to soil particles and do not interact with the bacteria, nor do we know the concentration of streptomycin required to inhibit the growth of almost all soil bacteria. Therefore, before we can discuss the relevance of the concentration of antibiotic added to soil, we first need to develop methods and breakpoints of relevance for this environment (Walsh, 2013). Such techniques and guidelines are vital to assess the impact of antibiotics on the bacterial populations in environments outside of clinically relevant pathogens.

Our study showed no significant difference due to pH for individual taxa, including the intrinsically resistant bacteria belonging to the *Pseudomonas*, *Burkholderia*, and *Stenotrophomonas* species. These results are in contrast to previous 16S rRNA amplicon sequencing studies, where Actinobacteria and Bacteroidetes showed correlation to high pH (Nacke et al., 2011). Our results are also in contrast to previous studies, as Acidobacteria abundances did not decrease with soil pH nor did the abundances of Actinobacteria and Bacteroidetes positively correlate with soil pH and the bacterial diversity was not associated with pH (Shange et al., 2012). The changes in the abundance of individual taxa due to treatment i.e., streptomycin or water and pH were not statistically significant.

**Figure 3:**
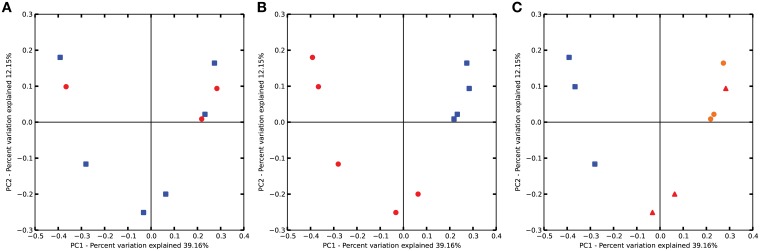
**Principal component analysis (PCoA) of bacterial communities as affected by (A) Treatment of streptomycin or water, (B) soil pH, and (C) soil site orchard**. Plots are based on Bray-Curtis dissimilarities comparing bacterial communities according to treatment, soil pH or orchard. *P*-values were calculated using PERMANOVA. **(A)** Circle, treated with streptomycin; square, treated with water; **(B)** circle, low pH; square, neutral pH; **(C)** triangle, Güttingen orchard; circle, Wädenswil orchard; square, Lindau orchard.

The entire communities of bacteria were analyzed for variations in bacterial abundance using ADONIS, ANOSIM, MRPP, and PERMDISP algorithms provided by the QIIME software suite. The results indicate no differences among streptomycin and water treated plots (Figure 3A). There are strong differences (*p* < 0.001) due to pH (low vs. neutral) (Figure 3B). Thus, although the soil use and management strategies were the same in each of the orchards, the soil pH had a stronger influence over the bacterial population than the management practices, such as the addition of streptomycin. The phylogenetic relationships of the bacterial communities cluster the three Lindau soils together and separately from the remainder of the soils (Figure 3C). The remaining two clusters consist of the Wädenswil and Güttingen orchards together. Soils derived from identical management systems, i.e., apple orchards under the same management practices do not necessarily harbor the same bacterial communities.

There was no significant difference in the bacterial populations associated with streptomycin use in the three orchards over time. The bacterial populations were also stable within the orchards over 3 years of treatment with streptomycin and thus there was no cumulative effect of streptomycin treatment for 3 years. Previous studies on the effects of antibiotic use in agriculture have frequently focused on cultured bacteria or resistance genes (Tolba et al., 2002). Culture-based approaches are limited to a small fraction of the entire soil bacteria and analysis of specific resistance genes provides information on the influence of streptomycin on the resistant population. However, it is also important to identify the influence of streptomycin on antibiotic susceptible bacteria and unculturable bacteria. Our study analyzed the entire bacterial community within soil and compared soils from the same orchard and same management practices to identify if there were differences, which were associated with streptomycin treatment.

In 2009, the American Academy for Microbiology compiled a report discussing antibiotic resistance and the factors that influence the development and spread of resistance calling for more information on the impact of streptomycin use in agriculture (American Academy of Microbiology, 2009). Our study has identified that neither specific taxa abundances nor the entire bacterial population abundances were altered by treatment with streptomycin. This study has also identified that the use of streptomycin in apple orchards did not significantly alter the bacterial diversity in the soils and does not have an adverse effect on the bacterial populations of treated soils. The strongest differences in entire populations were due to pH and site. The phylogenies of the bacterial communities from the same orchard did not necessarily cluster together, suggesting that variations in soil bacterial population were not influenced by land use. These results are in contrast to the studies of grassland and forest land-use and different management strategies, which have previously been found to influence the fungal and bacterial diversity and composition (Birkhofer et al., 2012; Shange et al., 2012).

In contrast to previous studies, our data identified differences only in the abundances of specific phyla Acidobacteria, Alphaproteobacteria, and Deltaproteobacteria when the Lindau orchard soil populations, with a low pH, were compared to the other two orchards soil populations with a neutral soil pH. No differences in the abundances of other specific taxa were associated with soil pH (Will et al., 2010; Nacke et al., 2011). We have however, identified strong differences in the abundances of the entire bacterial communities due to pH indicating that the individual taxa abundance changes are too low to be statistically significant alone but these small changes are only noticeable at the entire population level when taken together.

## Conclusions

The use of streptomycin as part of the agricultural land management did not influence the bacterial abundance or bacterial diversity within these soils. Our study was performed using replicated treated and control orchards, which are vital for statistical analyses. We conclude, similar to a study on the effect of streptomycin on the bacterial community in apple tree leaf samples and orchard soil samples from the US that the use of streptomycin did not have a detrimental effect on the bacterial population of the soil (Yashiro and McManus, 2012; Shade et al., 2013). There were no significant increases in the abundance of the intrinsically resistant bacteria belonging to the *Pseudomonas*, *Burkholderia*, and *Stenotrophomonas* species associated with the use of streptomycin. This study contributes to the increasing scientific evidence, which suggests that the use of streptomycin in apple orchards has a low impact on the bacterial ecosystem.

## Author contributions

Fiona Walsh designed the experiments, performed the sampling, DNA extractions and data analysis, and wrote the manuscript, Sarah M. Owens performed the 16S sequencing and contributed to the manuscript writing, Jürg E. Frey and Daniel P. Smith performed the data analysis and contributed to the manuscript writing. Sample processing, sequencing, and core amplicon data analysis were performed by the EMP (www.earthmicrobiome.org) and all amplicon and meta data has been made public through the data portal (www.microbio.me/emp). Work was conducted within the European research network COST TD0803 Detecting evolutionary hotspots of antibiotic resistances in Europe (DARE). The authors thank Professor John Bunge for his advice and assistance with the data analysis and statistics. The results of this study were presented at the Swiss Society for Microbiology meeting in Interlaken, June 2013. Brion Duffy contributed to results interpretation and manuscript preparation.

## Funding

This project was funded by the Swiss Federal Office for Agriculture, the Swiss Federal Office for the Environment and the Swiss Expert Committee for Biosafety (SECB). The funding source played no role in the design, collection and analyses of the data.

## Supplementary material

The Supplementary Material for this article can be found online at: http://www.frontiersin.org/journal/10.3389/fmicb.2013.00383/abstract

## Conflict of interest statement

The authors declare that the research was conducted in the absence of any commercial or financial relationships that could be construed as a potential conflict of interest.
