# Supplementary material for: Restricted streptomycin use in apple orchards did not adversely alter the soil bacteria communities
Source: Front Microbiol. 2014 Jan 31;4:383. doi: 10.3389/fmicb.2013.00383 (PMC3908321; doi:10.3389/fmicb.2013.00383)
Supplement: Supplementary file 1 [file DataSheet1.DOCX]

Table S1. The statistically significant variations between the abundance of all taxa between the different soils using the student t-test

| Category1 | Category2 | P-Value | Average1 | Average2 | PctChange | TaxaLevel | TaxaID |
| --- | --- | --- | --- | --- | --- | --- | --- |
| Lindau_Apple_Orchard | non-Lindau_Apple_Orchard | 1.41E-04 | 33 | 370 | 11% | Class | Acidobacteria-6 |
| Lindau_Apple_Orchard | Wadenswil_Apple_Orchard | 4.25E-06 | 327 | 6 | 11% | Order | Acidobacteriales |
| Lindau_Apple_Orchard | Wadenswil_Apple_Orchard | 4.25E-06 | 327 | 6 | 11% | Class | Acidobacteria |
| Lindau_Apple_Orchard | non-Lindau_Apple_Orchard | 2.13E-08 | 327 | 20 | 10% | Order | Acidobacteriales |
| Lindau_Apple_Orchard | non-Lindau_Apple_Orchard | 2.13E-08 | 327 | 20 | 10% | Class | Acidobacteria |
| Lindau_Apple_Orchard | non-Lindau_Apple_Orchard | 0.0003556 | 31 | 330 | 10% | Order | iii1-15 |
| Lindau_Apple_Orchard | non-Lindau_Apple_Orchard | 0.00041778 | 615 | 339 | 9% | Class | Alphaproteobacteria |
| Lindau_Apple_Orchard | non-Lindau_Apple_Orchard | 1.72E-04 | 66 | 299 | 8% | Class | Deltaproteobacteria |
| Lindau_Apple_Orchard | non-Lindau_Apple_Orchard | 6.42E-04 | 24 | 256 | 8% | Family | blank |
| Lindau_Apple_Orchard | non-Lindau_Apple_Orchard | 9.54E-05 | 175 | 13 | 5% | Family | Koribacteraceae |
| Lindau_Apple_Orchard | non-Lindau_Apple_Orchard | 0.0008879 | 14 | 152 | 5% | Order | Syntrophobacterales |
| Lindau_Apple_Orchard | non-Lindau_Apple_Orchard | 0.00013412 | 141 | 7 | 4% | Family | Acidobacteriaceae |
| Lindau_Apple_Orchard | non-Lindau_Apple_Orchard | 4.99E-07 | 134 | 16 | 4% | Family | blank |
| Lindau_Apple_Orchard | non-Lindau_Apple_Orchard | 4.99E-07 | 134 | 16 | 4% | Order | Ellin329 |
| Lindau_Apple_Orchard | non-Lindau_Apple_Orchard | 3.38E-04 | 103 | 7 | 3% | Family | blank |
| Lindau_Apple_Orchard | non-Lindau_Apple_Orchard | 3.38E-04 | 103 | 7 | 3% | Order | blank |
| Lindau_Apple_Orchard | non-Lindau_Apple_Orchard | 3.38E-04 | 103 | 7 | 3% | Class | Acidobacteria-2 |
| Lindau_Apple_Orchard | non-Lindau_Apple_Orchard | 0.00113416 | 138 | 41 | 3% | Family | Solibacteraceae |
| Lindau_Apple_Orchard | non-Lindau_Apple_Orchard | 0.00113416 | 138 | 41 | 3% | Order | Solibacterales |
| Lindau_Apple_Orchard | non-Lindau_Apple_Orchard | 1.13E-03 | 138 | 41 | 3% | Class | Solibacteres |
| Lindau_Apple_Orchard | non-Lindau_Apple_Orchard | 5.18E-05 | 105 | 16 | 3% | OTU | 13851 |
| Lindau_Apple_Orchard | non-Lindau_Apple_Orchard | 6.77E-05 | 137 | 59 | 3% | Family | Hyphomicrobiaceae |
| Lindau_Apple_Orchard | non-Lindau_Apple_Orchard | 4.11E-04 | 103 | 31 | 2% | Class | Gemmatimonadetes |
| Lindau_Apple_Orchard | non-Lindau_Apple_Orchard | 1.18E-03 | 127 | 69 | 2% | Order | Rhodospirillales |
| Lindau_Apple_Orchard | non-Lindau_Apple_Orchard | 0.00034722 | 52 | 4 | 2% | Family | blank |
| Lindau_Apple_Orchard | non-Lindau_Apple_Orchard | 4.05E-04 | 49 | 3 | 2% | OTU | 10839 |
